# Does bird metabolic rate influence mosquito feeding preference?

**DOI:** 10.1186/s13071-018-2708-9

**Published:** 2018-02-23

**Authors:** Jiayue Yan, Juli Broggi, Josué Martínez-de la Puente, Rafael Gutiérrez-López, Laura Gangoso, Ramón Soriguer, Jordi Figuerola

**Affiliations:** 1 Estación Biológica de Doñana (EBD-CSIC), Avda. Américo Vespucio, 26, E-41092 Seville, Spain; 2 CIBER Epidemiología y Salud Pública (CIBER ESP), Seville, Spain

**Keywords:** Mosquito blood-feeding, Resting metabolic rate, Host attractiveness, Host body mass, Host defensive behaviour, Intraspecific difference

## Abstract

**Background:**

Host selection by mosquitoes plays a central role in the transmission of vector-borne infectious diseases. Although interspecific variation in mosquito attraction has often been reported, the mechanisms underlying intraspecific differences in hosts’ attractiveness to mosquitoes are still poorly known. Metabolic rate is related to several physiological parameters used as location cues by mosquitoes, and so potentially affect host-vector contact rates. Therefore, individual hosts with higher metabolic rates should be more attractive to host-seeking mosquitoes. Here, we experimentally investigated the role of bird metabolic rate in the feeding preferences of *Culex pipiens* (Linnaeus), a widespread mosquito vector of many pathogens affecting human and wildlife health.

**Results:**

*Passer domesticus* (Linnaeus) pairs containing one bird treated with 2,4-dinitrophenol (DNP) and the other injected with phosphate-buffered saline solution (PBS) (i.e. control) were simultaneously exposed overnight to mosquitoes. The treatment did not affect the proportion of mosquitoes biting on each individual. However, mosquito feeding preference was negatively associated with bird resting metabolic rate but positively with bird body mass. These two variables explained up to 62.76% of the variations in mosquito feeding preference.

**Conclusions:**

The relationships between mosquito feeding preferences and individual host characteristics could be explained by enhanced anti-mosquito behaviour associated with higher metabolic rates. The potential role of cues emitted by hosts is also discussed. Thus, individuals with high metabolism may actively avoid being bitten by mosquitoes, despite releasing more attractant cues. Since metabolic rates can be related to individual differences in personality and life history traits, differences in mosquitoes’ feeding preferences may be related to intraspecific differences in exposure to vector-borne pathogens.

**Electronic supplementary material:**

The online version of this article (10.1186/s13071-018-2708-9) contains supplementary material, which is available to authorized users.

## Background

Mosquitoes (Diptera: Culicidae) are responsible for the transmission of multiple vector-borne pathogens that cause diseases such as malaria, West Nile fever and yellow fever [1]. Host selection by mosquitoes is recognized as a key factor affecting pathogen amplification and transmission risk since it drives host-vector contact rates [2, 3]. Differential mosquito biting preferences have been reported at host interspecific level [3–8], but also among individuals within species [9–11]. Recent studies demonstrated that host characteristics such as body size, age, sex, reproductive status, breath constituents, and health status are proximate factors driving variations in individual host attractiveness to mosquitoes [10–15]. These studies hypothesized that mosquito feeding preferences may be driven by host cues including CO_2_ plumes, odours, sweat and movements, which are often associated with host metabolism. Understanding the individual characteristics underlying these asymmetries in mosquito blood-feeding is of great importance as variability in contact rates could result in heterogeneous individual risk for pathogen transmission [16, 17]. However, the mechanisms underlying host intraspecific differences in mosquito attraction are still poorly understood [16].

Mosquitoes use visual, thermal and chemical cues to detect their hosts [18]. Metabolic rates of host animals are related to the activity and physiology of an individual [19]. This in turn is directly linked to the emission of CO_2_, heat and humidity [20], which may enhance mosquito attraction [21]. On the other hand, metabolic rate may also be associated with defensive behaviour, with individuals of higher metabolism being more restless and hence more difficult for mosquitoes to bite. In addition, body mass (BM) is usually a positive correlate of metabolic rate in many organisms [22]. In birds, metabolic rate is positively associated with BM as supported by a study on 231 species [23], but a negative association was also reported within small-sized hummingbirds [24]. Owing to the potential link with metabolic rate, BM may also affect the emission of multiple host-seeking cues as well as the defensive behaviour, which may affect mosquito attraction. A positive link between BM and blood-sucking insect attraction has been reported by a high number of authors working with groups of arthropod vectors such as mosquitoes [25, 26], biting midges *Culicoides* [27] and blackflies [28, 29]. However, the specific role of avian BM in mosquito attraction at intraspecific level remains poorly studied. Finally, a potential bias in studies of mosquito preference is that much research to date has focused on mosquito attractants and whether or not one particular individual will be chosen as a host before another one. However, under natural conditions individuals exposed to mosquito bites are in many cases surrounded by other potential hosts. Therefore, the likelihood of being bitten may not only depend on these attractant factors but also on the host composition, that is, whether or not they are surrounded by more attractive and/or susceptible counterparts (i.e. the infection intensity by blood parasites) [15].

Despite the potential importance of host metabolic rate in mosquito feeding preferences, to the best of our knowledge no study has yet experimentally tested the link between host metabolic rate and mosquito host selection. To do that, we assigned house sparrows *Passer domesticus* (Linnaeus) to two experimental treatments: birds injected with 2,4-dinitrophenol (DNP) or treated as controls, and exposed them to the bites of the mosquito *Culex pipiens* (Linnaeus) in pairs. *Culex pipiens* is widespread and acts as the main bridge vector for a number of pathogens affecting humans and wildlife including house sparrows (e.g. *Haemoproteus* [30], *Plasmodium* [31], West Nile virus and Saint Louis encephalitis virus, reviewed in [6]). Our experimental approach simulates a situation of host selection where birds with different physiological conditions grouped together during the breeding season or at roosts, and mosquitoes are active in seeking hosts.

Here we tested the hypothesis that mosquitoes preferentially bite individuals with higher metabolic rates, as they may release more attractant cues for host-seeking mosquitoes. Alternatively, they will be bitten less than their counterparts with lower metabolic rates, as other factors related to bird metabolism, such as anti-mosquito behaviour, may overrule the effect of attractant cues on determining mosquito bites.

## Methods

### Mosquito rearing

Mosquito larvae were collected from Cañada de los Pájaros (Seville, Spain) in summer 2014 and were reared in plastic trays containing water in climatic chambers. Larvae were supplied with shrimp food (Mikrozell 20 ml/22 g; Dohse Aquaristik GmbH & Co. KG, D-53501, Gelsdorf, Germany). Mosquitoes were kept at 27 (±1) °C and 65–70% relative humidity (RH) under a photoperiod of 12:12 h (Light:Dark). One to 5 days after emergence, adult mosquitoes were anaesthetized with diethyl ether [32] and then sexed and identified to species level [33] on chilled Petri dishes using a stereomicroscope (Nikon SMZ-645, Tokyo, Japan). Female *Cx. pipiens* were retained and placed in insect-rearing cages (BugDorm-43030F, 32.5 × 32.5 × 32.5 cm; MegaView Science, Taiwan) in the same chamber conditions as above with ad libitum access to 1% sugar solution until 10–19 days-old. Mosquitoes were deprived of the sugar solution 24 h prior to the experiment and maintained with water until 12 h before the experiment.

### Bird sampling and maintenance

Thirty juvenile house sparrows were trapped in Huelva province (southern Spain) in July 2014 using mist nets. We chose wild house sparrows as vertebrate hosts because this species is a natural reservoir for multiple vector-borne pathogens [34–36] and has been reported to be one of the preferred hosts of several mosquito species including *Cx. pipiens* [7, 37]. Yearling birds were individually marked with metal rings and weighed with a digital scale (Pesola-MS500, Pesola©, Switzerland). A small blood sample from each bird was taken using jugular venipuncture for future molecular analyses (see below). Subsequently, birds were transported to the Animal Experimentation Unit at the Estación Biológica de Doñana (EBD-CSIC) and kept in pairs in cages (58.5 × 25 × 36 cm) in a vector-free room at 22 ± 1 °C and a 12 L:12D photocycle. Water and food (mixed grain) were provided ad libitum. Two to 5 days after finishing the experiment, birds were released at the site of capture.

### Measurements of bird resting metabolic rates and body mass

The 30 house sparrows used in this study consisted of 21 males (10 DNP and 11 control) and 9 females (5 DNP and 4 control). Of the 15 pairs, 9 contained a male and a female bird and 6 pairs included 2 male birds.

The RMR of each bird was measured as the minimum oxygen consumption under post-absorptive digestive conditions during its resting cycle [38, 39]. RMR was measured during a 12 h period from 20:00 h to 08:00 h using an open-circuit respirometer (Sable Systems International, Las Vegas, NV, USA). Oxygen consumption (ml O_2_/min) was estimated as the lowest value of the averages of 10 min runs [40]. Birds’ BM was recorded before RMR measurements were taken.

### Bird treatments and blood-feeding assays

The night following the RMR measurements, half of the birds (*n* = 15) were randomly injected subcutaneously with 0.2 mg of DNP diluted in 0.04 ml of phosphate-buffered saline solution (PBS) (DNP group), while the remaining birds (*n* = 15) were injected with the same volume of PBS (control group). DNP is an artificial decoupler of oxidative phosphorylation [41] and, acting as a protonophore, facilitates the leak of the protons that build up the force to drive ATP synthesis and results in poor connection between oxidation and phosphorylation. This induces an increase in the metabolic rate (i.e. oxygen consumption) to compensate for mitochondrial inefficiency and to meet energy demands [42]. Immediately after injection, a pair of birds consisting of a DNP and a control bird was exposed to 10–19 days-old unfed female mosquitoes in the dark for 12 h from 20:00 h to 08:00 h (activity peak of *Cx. pipiens*, see [43, 44]). In all, 15 trials over 3 nights were conducted. In each trial, a birdcage (38.5 × 26 × 5.5 cm) containing a pair of birds was exposed to an average of 190 (range: 181–198) unfed *Cx. pipiens* females in insect-rearing tents (BugDorm-3120, white, 60 × 60 × 60 cm). Mosquitoes and birds were allowed to move and come into contact without any restrictions, as mosquitoes were able to freely enter the birdcages. At the end of each trial, blood-engorged mosquitoes were aspirated from inside tents, counted and stored at -20 °C.

The RMR could not be measured immediately following DNP and PBS injection because the mosquito exposure trials were taking place. Thus, in order to assess the effect of DNP injection on bird RMR, we captured eight additional house sparrows; four of them were injected with DNP and the other four with PBS as in the previous experiment. Immediately after the injection, the RMR of these individuals was recorded during 12 h using the same approach reported above.

### Molecular assays

We isolated genomic DNA from blood samples taken from birds using the DNA Kit Maxwell® 16LEV (Promega, Madison, WI, USA) [45]. Birds were molecularly sexed and their *Plasmodium*, *Haemoproteus* and *Leucocytozoon* infection status were determined [46]. To reduce any potential effect of host infection status on mosquito host selection (see [12, 15]), only birds without detectable infection by these parasites were included in the experimental procedure.

Thirty engorged mosquitoes were randomly selected after each trial to determine the origin of their blood meals; the only exception was one trial that produced only nine engorged mosquitoes, which were all analysed. Mosquito abdomens were separated from the head-thorax using sterile pipette tips and Petri dishes on an ice surface. Genomic DNA of the blood meal was isolated using the HotSHOT procedure (see [47, 48]). DNA samples were stored at -20 °C until PCR amplification analyses.

The sex of birds bitten by each mosquito was determined from the blood meal [49, 50]. We used the primer pair P2 (5′-TCT GCA TCG CTA AAT CCT TT-3′) and P8 (5′-CTC CCA AGG ATG AGR AAY TG-3′) that targets the sex-related chromo-helicase-DNA-binding gene (*CHD*). PCR amplification was carried out in a total volume of 25 μl in thermal cyclers (BIO-RAD T100, Hercules, USA; and Agilent Sure Cycler 8800, Santa Clara, USA). The reaction conditions and cycle temperatures are described in [50]. Positive amplifications were visualized in 3% agarose gels. This procedure was used to partially identify the origin of the mosquito blood meals. In particular, for the nine pairs containing a male and a female bird, the blood meals with one-band amplification were identified as male-derived blood meals. Blood meals providing two bands of amplification were identified as the blood meals taken from female birds exclusively or as the mixed blood meals taken from both male and female birds.

These two-band samples and those from six bird pairs including two males were processed using eight different primer pairs to target different microsatellite fragments of the genotyped birds (see Additional file 1: Table S1 [51]). Microsatellite amplifications were conducted with a total volume of 20 μl for each sample containing 2 μl of extracted DNA sample, 2 μl of PCR buffer (10×), 0.6 μl of MgCl_2_ (50 mM), 0.16 μl of dNTPs (25 mM), 0.1 μl of Taq, 13.54 μl of H_2_O and 0.8 μl of primer for two DNA strands, respectively. Positive amplifications were visualized in 3% agarose gels to identify homozygous (one band) and heterozygous (two bands) individuals for each microsatellite and compared between birds from the same trial pair.

For pairs composed of two males, we selected pairs of microsatellite primers having mutually exclusive amplification patterns for each bird of the pair. This procedure allows birds to be identified and reduce the cost of sequencing. Samples with one-band amplification for either of the pair of primers were identified as blood meals from either one of the pair of birds, while two-banded amplifications for the two microsatellites were identified as mixed-blood meals. For those cases where birds showed a similar amplification pattern, we sequenced four different microsatellites (Pdo A08, B01, D09 and F09; see also Additional file 1: Table S1) from bird blood samples and mosquito blood meals using the 3130*xl* ABI Genetic Analyzer (Applied Biosystems, Foster City, USA). Alleles were scored using GENEMAPPER v.3.7 (Applied Biosystems). The origin of the remaining samples was resolved by comparing the size of alleles amplified by multiple primer pairs. Consequently, for each trial we obtained the number of mosquitoes that had bitten each individual and the number of mosquitoes that had bitten both birds. To assess the reliability of the assignment of blood origin, both the sex determination and microsatellite genotyping were run in duplicate for 52 and 12 samples, respectively. No inconsistent results were found.

### Statistical analyses

The RMR in the DNP and control groups prior to the experimental injection was compared with Generalized Linear Models (GLMs) with normally distributed errors. The same procedure was also used to test for differences in RMR between control and DNP birds immediately after injection. Generalized Linear Mixed Models (GLMMs) with binomial error and logit link function were used to test the potential differences in the number of fed mosquitoes in relation to total introduced mosquitoes (included as the binomial denominator) between heterogeneous (containing one male and one female bird) and homogenous (containing two males) trials. We also used GLMMs to test the relationship between bird RMR and mosquito feeding preference in the trials. In this case, the number of engorged mosquitoes on a particular bird was analysed as a binomial variable with the total number of engorged mosquitoes as the binomial denominator. This response variable ‘feeding preference’ was incorporated into models codified as the number of mosquitoes that fed on one bird with respect to the number of mosquitoes that fed on the other bird within a pair (without mixed blood meals) using the *cbind* function. Before fitting models, bird RMR was logistic-transformed to attain normality [52]. In the models, BM and RMR were introduced as covariates; bird sex, treatment, the interaction between sex and treatment and between treatment and RMR were incorporated as explanatory factors; and bird identity was included as a random factor in order to cope with the overdispersion found in models with a count response [53]. In addition, bird pair was also included as a random factor as some pairs were composed of birds of different gender and so direct comparisons between pair members could not be conducted without controlling for confounding variables such as bird sex and BM. The multi-collinearity of explanatory variables was first assessed by calculating the generalized variance inflation factors (gVIFs) and, as these gVIF values were < 4 for the two continuous variables, both were incorporated into further analyses [54]. Model selection was based on the second order Akaike’s information criteria (AICc). Delta AICc (ΔAICc) was calculated as the difference in AICc between the model with the lowest AICc and other models.

First, we fitted a global model containing all the predictors using the *lme4* package v.1.1 [55]. We standardized input variables before model analysis using the *arm* package v.1.8 [56]. We then derived a set of sub-models (including the null model, which contained only the intercept) from the global model by using the dredge function implemented in the *MuMIn* package v.1.15 [57]. A ‘top model set’ was created by selecting those models with a difference of ΔAICc < 2. If more than one model was selected in the top model set, we performed a model-averaging approach to summarize the results using the *MuMIn* package v.1.15 [58]. Finally, as a measure of goodness-of-fit for mixed models, we calculated the explained variance (conditional *R*^2^) for each of the selected top models [58]. All analyses were carried out in R software v.3.2.5 [59].

## Results

The percentage of fed mosquitoes in relation to total introduced mosquitoes in each trial varied from 5.46 to 40.6% (mean ± SE = 27.2 ± 2.51%; see also Additional file 2: Table S2). The number of fed mosquitoes between heterogeneous (containing one male and one female bird) and homogenous (containing two males) trials did not differ significantly (estimate ± SE = 0.358 ± 0.282, *z* = 1.142, *P* = 0.25). The blood meal origin of 429 mosquitoes was identified to the individual level. The mean (± SE) number of mosquitoes with a blood meal derived from a single individual was 12.63 ± 1.74 (range: 0–30; Additional file 2: Table S2). An average of 3.33 (range: 0–7) mosquitoes contained mixed blood meals per trial. In subsequent analyses we only present the results excluding these mixed blood meals as results including this data were qualitatively the same (data not shown).

**Fig. 1:**
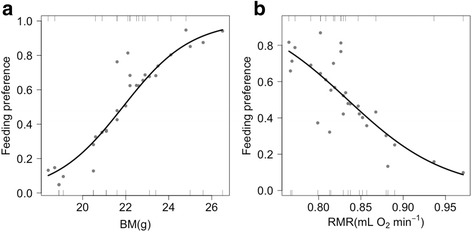
Relationship between mosquito feeding preferences and body mass (BM) (**a**) and resting metabolic rate (RMR, logistic transformation) (**b**). The blood meal origin was determined from 429 engorged mosquitoes. The total sample size of house sparrows was 30, with 15 replicates for control and DNP groups, respectively. Estimates were derived from the highest-ranked models according to the AICc. Each conditional relationship was plotted by holding the median value of the other variable using the *visreg* package (version 2.2.2) in R

Prior to the treatment, the resting metabolic rate (RMR) did not differ significantly between birds assigned to the DNP and control groups (estimate ± SE = -0.001 ± 0.017, *t*_(29)_ = -0.027, *P* = 0.98). Two top models (Table 1) were selected according to the AICc criterion. The explained variance (conditional *R*^2^) was 62.76% (model with BM and RMR (logistic-transformed) as explanatory variables) and 54.84% (model with only BM). Neither of these models included the experimental treatment and the treatment had no significant effect when added to the model with the lowest AICc (estimates ± SE = -0.086 ± 0.766, *z* = -0.112, *P* = 0.91). The averaged estimates indicated that feeding preference was positively associated with BM but negatively correlated to RMR (Table 2). The relative importance of BM and RMR was 1.00 and 0.63, respectively. The 95% confidence intervals for the parameter estimates did not include zero, indicating that these two predictors significantly influenced feeding preference (Table 2, Fig. 1). These results were not an artefact caused by collinearity between both variables since the correlation coefficient between RMR and BM was very low and not significant (*r* = 0.223, *P* = 0.235), and the results did not change qualitatively when using the residuals of RMR against BM as a predictor instead of RMR (results not shown).

**Table 1:** Model selection from the set of GLMMs analyzing the variation in mosquito feeding preferences. All models include bird pair and bird identity as random terms. The variables included in each model are represented by +. Top models are marked in bold

BM	Sex	RMR	T	Sex*T	RMR*T	AICc	ΔAICc	ω AICc
**+**		**+**				**194.7**	**0.00**	**0.364**
**+**						**195.8**	**1.10**	**0.210**
+	+	+				197.9	3.12	0.077
+		+	+			197.9	3.14	0.076
+	+					198.5	3.75	0.056
+			+			198.7	3.97	0.050
+		+	+		+	199.2	4.42	0.040
						199.7	4.95	0.031
+	+	+	+			201.3	6.55	0.014
		+				201.3	6.58	0.014
+	+		+			201.6	6.86	0.012
	+					201.6	6.91	0.012
			+			201.9	7.14	0.010
+	+	+	+		+	202.7	7.92	0.007
+	+	+	+	+	+	202.9	8.15	0.006
+	+	+	+	+		203.2	8.42	0.005
		+	+			203.7	8.93	0.004
	+	+				203.9	9.12	0.004
	+		+			203.9	9.19	0.004
+	+		+	+		204.3	9.60	0.003
		+	+		+	206.0	11.25	0.001
	+	+	+			206.4	11.64	0.001
	+		+	+		206.5	11.74	0.001
	+	+	+	+		208.8	14.10	0.000
	+	+	+		+	209.2	14.43	0.000
	+	+	+	+	+	211.3	16.61	0.000

*Abbreviations*: *BM* body mass, *Sex* bird sex, *RMR* resting metabolic rate (logistic transformation), *T* treatment, sex*T interaction between sex and treatment, RMR*T interaction between RMR and treatment, *∆AICc* [AICc*i* –min AICc]

**Table 2:** Summary statistics of the averaged model explaining the variation in feeding patterns of *Cx. pipiens*. Significant effects are highlighted in bold

Explanatory variable	Estimate	SE	*z*-value	95% CI		*P*
Intercept	-0.033	0.384	0.081	-0.240	1.014	0.936
BM	2.453	0.835	2.804	0.608	3.311	**0.005**
RMR	-1.612	0.777	1.974	-2.533	-0.003	**0.048**

*Abbreviations*: *BM* bird body mass, *RMR* resting metabolic rate (logistic transformation), *SE* standard error, *CI* confidence interval

In order to identify causes explaining the non-significant effect of the experimental treatment, we measured the RMR of eight birds immediately after the injection with DNP (*n* = 4) or treated as controls (*n* = 4). The RMR of birds did not statistically differ between the two experimental groups (estimate ± SE = -0.073 ± 0.245, *t*_(7)_ = -0.297, *P* = 0.78).

## Discussion

In this study we tested the relationship between host metabolic rate and the feeding preference of *Cx. pipiens* mosquitoes. We found that these mosquitoes preferred to feed on birds with higher BM but lower RMR. To date this is the first evidence that host metabolic rate does affect mosquito feeding preference. As our focal species *Cx. pipiens* is an important vector for multiple infectious diseases, identifying factors affecting the biting preferences of this mosquito species may throw some light on the epidemiology of these pathogens.

The positive association between feeding preference and BM suggests that larger individuals may release more cues that facilitate their detection and location by mosquitoes along multiple pathways such as vision, motion and odour. On the other hand, host breath may contain allomonal properties that potentially reduce mosquito attraction, as shown in human hosts with a wind-tunnel experiment by Mukanaba et al. [11]. In our case, however, birds and mosquitoes were kept in close proximity but under complete darkness and in a windless environment. Hence the role of visual cues is probably limited here and olfactory cues as well as heat and humidity from two birds may mix up and so may not serve for host discrimination but may rather indicate the specific body parts as biting sites for mosquitoes [21, 60]. In addition, rather than the number of attracted mosquitoes, our study measured mosquito host preference in term of the number of blood-fed mosquitoes, which may be subject to the influence of a host defensive response. In this context, motion (including anti-mosquito behaviour) rather than vision, odour or breath could be the most important factor determining mosquito feeding patterns. Nonetheless, we cannot rule out the possibility that the latter cues could also affect, at least in part, our results. Smaller individuals tend to move more frequently than larger ones [61], and avian defensive behaviour can greatly affect mosquito feeding success [62–65]. This could explain why birds with higher BM were bitten more frequently by mosquitoes. Another non-mutually exclusive explanation could be that larger individuals are an easier prey for mosquitoes since, when compared to smaller or more active (higher RMR) individuals, they may offer larger biting surfaces and be less proficient at avoiding bites. Our study adds to the large body of evidence showing a positive correlation between host body size/mass and the attraction of different insect including mosquitoes [25, 26], biting midges [27] and blackflies [28, 29] and highlights the importance of host size on mosquito blood-feeding at the intraspecific level.

Contrary to our prediction, birds with lower RMR suffered more mosquito bites than individuals with higher RMR. Higher metabolic rate is expected to be associated with the increased emission of the cues used by host-seeking mosquitoes [18]. Mosquito blood-feeding is a complex behaviour that includes different phases, from appetitive behaviour to a consummatory reaction and the cessation of feeding [66]. In our study, birds were placed close (within 60 cm) to mosquitoes, and initially the heat and humidity released by hosts may have been used as clues for the detection by mosquitoes [21, 67]. However, after approaching their hosts, the success of blood-feeding is largely determined by bird behaviour since mosquitoes avoid those individuals/species that are more active at the time of biting [62]. In our study, birds were able to move freely during the exposure to mosquitoes and hence may have performed anti-mosquito behaviour to protect themselves from bites. The defensive behaviour displayed by birds against mosquitoes (i.e. foot stomping, head and wing movement, tail shaking [63]) may reduce the ability of mosquitoes to complete a blood meal [63–65] but are also energetically costly [67]. Animals with high RMR may be more active, aggressive, explorative and bold, while their low RMR counterparts may be calmer and shyer and have the tendency to avoid novel situations [68]. In this context, although birds with higher metabolic rates might have attracted more mosquitoes due to a greater emission of host location cues, the final feeding number of mosquitoes on this type of birds could be lower than birds with lower metabolic rates that may perform less intense anti-mosquito behaviour. Potential differences in anti-mosquito behaviours between bird classes, could explain results from [69] who found that adult pigeons (*Columba livia*) attracted more mosquitoes than juvenile individuals, but mosquito feeding success (proportion fed) was greater on juveniles than adult birds, Therefore, intensive movements powered by higher RMR could explain why mosquitoes bite preferably birds with lower RMR. Nonetheless, we did not perform direct observation of host anti-mosquito behaviour during night exposure to mosquitoes, and so the effect of host defensiveness on mosquito feeding preference calls for further research.

Despite the initial differences in RMR, we did not find any significant effect of the experimental treatment on the mosquito feeding preference. The DNP administration did not significantly affect the RMR of birds, as no differences in RMR were found between DNP-treated and control birds during the following 12 h after injection. Previous studies have recorded an increase in metabolic rates as a result of mild mitochondrial uncoupling by DNP administration in species including invertebrates [70], amphibians [71], birds [72] and mammals [73]. However, the efficacy of DNP in most of the cases was very short in time [74]. DNP can be quickly eliminated from the organism, and, for example, within 24 h up to 98% of DNP have been eliminated in ducks and rabbits [75], and the metabolic rate returned to normal values a day after injection [76]. If the efficacy of treatment did not significantly affect the RMR of birds or/and the efficacy lasted only during the period before mosquitoes were able to bite birds, this could explain, at least in part, why no significant effects were found in this study. In addition, the efficacy of DNP on RMR of animals may depend on the dose [74], route of administration and experimental conditions, such as temperature [77]. The subcutaneous administration of DNP to birds in our study may have resulted in a slower release of drug into the blood than oral DNP administration, delaying the impact on bird metabolic rate. Even so, it is possible that the dose injected to birds was not enough to modify the bird RMR, as an oral dose of 5 mg/l of DNP was reportedly insufficient to noticeably affect the metabolic rate of zebra finches [78]. Further studies are necessary in order to identify the effective dose of DNP to modify the RMR of wild house sparrows without increasing mortality or producing long-term damages in bird health.

Our study represents mosquitoes’ choice between different hosts emitting different physiological and behavioural cues. Although a bird with higher metabolism may release more cues that attract mosquitoes, the final outcome of mosquito feeding patterns may also depend on the surrounding hosts, which could reduce the individual risk of being attacked [79, 80], i.e. the per capita bird exposure to infected mosquitoes may be less given the encounter-dilution effect [81].

## Conclusions

Hosts’ metabolic rates and body mass may influence mosquito feeding preference at intraspecific host level. As metabolism is closely related to individual differences in personality [68], behaviour [67] and life history traits [72], these findings may have important implications for individual exposure to mosquito bites and consequently for the amount of exposure to vector-borne diseases.

## Additional files

Additional file 1: **Table S1.** Primers used in this study for genotyping of house sparrows. *Primers used in DNA sequencing; ^+^Primer labelled with FAM, VIC, NED or PET; adapted from Garnier et al. [51]. (DOC 35 kb)

Additional file 2: **Table S2.** Bird characteristics, experimental settings and mosquito blood-feeding data. *Abbreviations*: ID, bird identity; M/F, male/female; C/DNP, control/2,4-dinitrophenol; BM, body mass (g); RMR, resting metabolic rate (ml O_2_ min^-1^); TIM, total introduced mosquitoes (count); TFM, total fed mosquitoes (count); PTFM, percentage of total fed mosquitoes (%); TAM, total analysed mosquitoes (exclusively non-mixed blood meals, count); FM, fed mosquitoes (exclusively non-mixed blood meals, count); FP, feeding preference (ratio). (DOC 70 kb)

## Abbreviations

AICc: Akaike information criterion with a correction for finite sample sizes
ANOVA: Analysis of variance
ATP: Adenosine triphosphate
BM: Body mass
CHD: Chromo helicase DNA binding gene
CO_2_: Carbon dioxide
DNA: Deoxyribonucleic acid
DNP: 2,4-dinitrophenol
dNTP: Deoxynucleotide
GLMMs: Generalized linear mixed models
gVIF: Generalized variance-inflation factors
PBS: Phosphate buffered saline solution
PCR: Polymerase chain reaction
RMR: Resting metabolic rate
