# Supplementary material for: Does bird metabolic rate influence mosquito feeding preference?
Source: Parasit Vectors. 2018 Feb 23;11:110. doi: 10.1186/s13071-018-2708-9 (PMC5824498; doi:10.1186/s13071-018-2708-9)
Supplement: Supplementary file 1 — Table S1. Primers used in this study for genotyping of house sparrows. *Primers used in DNA sequencing; +Primer labelled with FAM, VIC, NED or PET; adapted from Garnier et al. [51]. (DOC 35 kb) [file 13071_2018_2708_MOESM1_ESM.doc]

**Additional file 1: Table S1.** Primers used in this study for genotyping of house sparrows. *Primers used in DNA sequencing; ^+^Primer labelled with FAM, VIC, NED or PET; Adapted from Garnier et al. [51].

| Locus name | Primer sequence (5´-3´) | GenBank Accession no. |
| --- | --- | --- |
|
| PdoA08* | AGCTTTTCAGGTCTCCTTCT+VIC CTACACCAGCAAGATCCATT | FJ422589 |
| PdoB01* | GCCTGCTTAAACTATCTTGG+PET GATATAGGGAGCAGAGTTCTTG | FJ422590 |
| PdoB04 | ATTTGGGTGGTTAGTTCAAA+FAM CAAATACAGTGCATCTACAACC | FJ422591 |
| PdoC11 | GCAGCATGTCATAATAGCAG+FAM TTTTCCTTTGCATACACCA | FJ422592 |
| PdoD09* | CTCTCCTGCTATGCTTCCT+PET CTTGGGATATGATGGAAATG | FJ422593 |
| PdoE09 | TGACTAAAATAGATCAAGGCTTTT+FAM TGCAAAGATACCAGAACTCAT | FJ422594 |
| PdoF05 | GCATATTTCTGGCATTCTTC+VIC TCAAATAAAGTGCTCCACAA | FJ422595 |
| PdoF09* | CACGGGTGGTATTTTATATG+NED ATGTTGCAGATTGAAAAGTG | FJ422596 |
