# Supplementary material for: Does bird metabolic rate influence mosquito feeding preference?
Source: Parasit Vectors. 2018 Feb 23;11:110. doi: 10.1186/s13071-018-2708-9 (PMC5824498; doi:10.1186/s13071-018-2708-9)
Supplement: Supplementary file 2 — Table S2. Bird characteristics, experimental settings and mosquito blood-feeding data. Abbreviations: ID, bird identity; M/F, male/female; C/DNP, control/2,4-dinitrophenol; BM, body mass (g); RMR, resting metabolic rate (ml O2 min-1); TIM, total introduced mosquitoes (count); TFM, total fed mosquitoes (count); PTFM, percentage of total fed mosquitoes (%); TAM, total analysed mosquitoes (exclusively non-mixed blood meals, count); FM, fed mosquitoes (exclusively non-mixed blood meals, count); FP, feeding preference (ratio). (DOC 70 kb) [file 13071_2018_2708_MOESM2_ESM.doc]

**Additional file 2: Table S2. Bird characteristics, experimental settings and mosquito blood-feeding data. *Abbreviations*: ID, bird identity; M/F, male/female; C/DNP, control/2,4-dinitrophenol; BM, body mass (g); RMR, resting metabolic rate (ml O_2_ min^-1^); TIM, total introduced mosquitoes (count); TFM, total fed mosquitoes (count); PTFM, percentage of total fed mosquitoes (%); TAM, total analysed mosquitoes (exclusively non-mixed blood meals, count); FM, fed mosquitoes (exclusively non-mixed blood meals, count); FP, feeding preference (ratio).**

| Bird pair | ID | Sex | Treatment | BM | RMR | TIM | TFM | PTFM | TAM | FM | FP |
| --- | --- | --- | --- | --- | --- | --- | --- | --- | --- | --- | --- |
| 1 | 1 | M | C | 20.6 | 1.71 | 183 | 10 | 5.46 | 9 | 3 | 0.33 |
|  | 2 | F | DNP | 18.4 | 1.18 |  |  |  |  | 6 | 0.67 |
| 2 | 3 | M | C | 25.6 | 1.58 | 181 | 41 | 22.65 | 25 | 6 | 0.24 |
|  | 4 | F | DNP | 25 | 1.2 |  |  |  |  | 19 | 0.76 |
| 3 | 5 | M | DNP | 21.1 | 1.62 | 185 | 41 | 22.16 | 27 | 6 | 0.22 |
|  | 6 | F | C | 22.6 | 1.4 |  |  |  |  | 21 | 0.78 |
| 4 | 7 | M | C | 22.2 | 1.5 | 186 | 59 | 31.72 | 28 | 27 | 0.96 |
|  | 8 | F | DNP | 19.1 | 1.19 |  |  |  |  | 1 | 0.04 |
| 5 | 9 | M | DNP | 20.5 | 1.72 | 186 | 67 | 36.02 | 28 | 4 | 0.14 |
|  | 10 | F | C | 22.2 | 1.88 |  |  |  |  | 24 | 0.86 |
| 6 | 11 | M | C | 23.1 | 1.64 | 191 | 41 | 21.47 | 23 | 13 | 0.57 |
|  | 12 | F | DNP | 20.9 | 1.44 |  |  |  |  | 10 | 0.43 |
| 7 | 13 | M | DNP | 21.6 | 2.69 | 196 | 70 | 35.71 | 25 | 5 | 0.20 |
|  | 14 | F | C | 24.1 | 1.58 |  |  |  |  | 20 | 0.80 |
| 8 | 15 | M | C | 22.9 | 3.51 | 194 | 64 | 32.99 | 25 | 9 | 0.36 |
|  | 16 | F | DNP | 26.5 | 2.09 |  |  |  |  | 16 | 0.64 |
| 9 | 17 | M | DNP | 23.4 | 1.79 | 198 | 30 | 15.16 | 27 | 6 | 0.22 |
|  | 18 | F | C | 23.5 | 1.51 |  |  |  |  | 21 | 0.78 |
| 10 | 19 | M | C | 18.9 | 1.38 | 197 | 80 | 40.61 | 26 | 0 | 0.00 |
|  | 20 | M | DNP | 22.1 | 1.56 |  |  |  |  | 26 | 1.00 |
| 11 | 21 | M | C | 21.6 | 1.48 | 189 | 34 | 17.99 | 28 | 7 | 0.25 |
|  | 22 | M | DNP | 18.7 | 1.22 |  |  |  |  | 21 | 0.75 |
| 12 | 23 | M | C | 22.5 | 1.6 | 190 | 58 | 30.53 | 28 | 22 | 0.79 |
|  | 24 | M | DNP | 22 | 1.99 |  |  |  |  | 6 | 0.21 |
| 13 | 25 | M | C | 22.8 | 1.33 | 191 | 74 | 38.74 | 24 | 20 | 0.83 |
|  | 26 | M | DNP | 21.1 | 1.75 |  |  |  |  | 4 | 0.17 |
| 14 | 27 | M | C | 24.8 | 1.56 | 192 | 58 | 32.21 | 26 | 26 | 1.00 |
|  | 28 | M | DNP | 20.5 | 2.01 |  |  |  |  | 0 | 0.00 |
| 15 | 29 | M | C | 18.9 | 1.47 | 192 | 52 | 27.08 | 30 | 0 | 0.00 |
|  | 30 | M | DNP | 21.6 | 1.4 |  |  |  |  | 30 | 1.00 |
